# Identifying vulnerable groups of community-dwelling older adults with a strong willingness to receive volunteer services based on machine learning methods

**DOI:** 10.1186/s12889-025-24902-7

**Published:** 2025-11-18

**Authors:** Lei Huang, Weihong Yang, Lina Wang, Peng Wang, Yan Lin, Yue Yao, Zhijie Zhao, Fengjian Zhang, Haiyan Zhang, Lulu Liao, Jie Hu, Yuqing Ye, Jinrong Yuan, Yilan Liu

**Affiliations:** 1 Cancer Hospital, The First Affiliated Hospital of Henan Medical University, Xinxiang, China; 2 School of Nursing, Henan Medical University, Xinxiang, China; 3 Quantitative Development, Queueco Limited, London, UK; 4 Department of Nursing, Union Hospital, Tongji Medical College, Huazhong University of Science and Technology, Wuhan, China

**Keywords:** Vulnerable groups, Community-dwelling, Older adults, Volunteer service, Machine learning

## Abstract

**Background:**

Although volunteer services play an increasingly important role in addressing the aging crisis, there is still insufficient evidence on which community-based older adult groups should be prioritized for service provision. This study aims to identify the key characteristics of vulnerable community-dwelling older adults with strong willingness and high demand for volunteer services.

**Methods:**

This study employed a cross-sectional descriptive design. A total of 852 community-dwelling older adults from four central cities in China were surveyed between March 25 and May 5, 2022, using a convenience sampling method. Data were analyzed using SPSS 26.0 and advanced machine learning techniques in Python.

**Results:**

Logistic regression (AUC = 0.975) and random forest (AUC = 0.970) achieved the best performance in predicting willingness, with the most critical predictors being absence of cohabiting family care and caregiving support, living alone, poor health and depressive status, having basic medical insurance, and advanced age. For demand level, multiple linear regression (R² = 0.230) performed best, identifying depression status, poor health, willingness to help others, and absence of government care as significant predictors. Model comparisons demonstrated robust and consistent variable importance rankings across algorithms.

**Conclusion:**

This study provides a scientific basis for developing more targeted and efficient volunteer service strategies, enabling closer alignment with older adults’ needs and more effective allocation of resources, thereby minimizing public resource waste. Its implementation has significant theoretical and practical value for optimizing service distribution.

**Supplementary Information:**

The online version contains supplementary material available at 10.1186/s12889-025-24902-7.

## Background

With the accelerating aging population, the proportion of people aged 60 and above globally has reached 12.3%, and is expected to rise to 22.0% by 2050 [1]. The aging process is particularly significant in China [2]. By the end of 2022, the population aged 60 and above had reached 280 million, accounting for 19.8% of the total population [3]. The most common pension model in China is the “9073” model, where 90% of older adults receive home-based care, 7% receive care in community settings, and 3% receive institutional care [4]. Although home-based care remains the preferred option, the traditional family care model has weakened due to changes in family structures [5]. Prior research has indicated that some older adults experience insufficient family support, which may lead to difficulties in daily living and health maintenance [6]. Meanwhile, as China enters a stage of accelerating population aging, the growing and diversifying needs of the older population pose increasing challenges to the existing social pension service system [7].

In this context, volunteer service refers to altruistic and unpaid activities aimed at helping others, and has become an important strategy for addressing the challenges of population aging. Unlike community health service personnel, who mainly provide health management and medical care, volunteers focus on offering companionship, emotional support, and basic assistance with daily activities. Recognizing the value of such contributions, the government has increasingly prioritized the development of volunteer services for older adults by improving mechanisms for social participation. In line with this, China’s “14th Five-Year Plan for National Aging Cause Development” proposes the establishment of a collaborative service model involving social workers, neighbors, volunteers, and healthcare providers to deliver care for older individuals in need [8].

Although volunteer services hold great potential in addressing population aging, existing research remains limited in scope, with most studies focusing on resource integration and the formulation of service strategies [9]. For example, regarding resource integration, Winterton et al. [10] suggested that community-based elderly services can be supported through diverse forms of volunteer participation, such as local hospitals, Red Cross organizations, nonprofit community groups, religious institutions, and small groups sponsored by larger organizations. Fullagar et al. [11] explored how professionals in health, sociology, and psychology can lead volunteer teams to deliver eldercare services, such as organizing health activities for older adults in communities. In terms of service strategies, Huang et al. [12] evaluated the initial motivations of volunteers with nursing backgrounds to participate in community-based eldercare and summarized the effective strategies they employed, including maintaining belief, knowing, being with, doing for, and enabling. Luger et al. [13] reported on the diversified services provided by non-professional volunteers to older adults in the community, such as home-based physical training, nutritional support, and social assistance to address malnutrition and frailty. Rantanen et al. [14] indicated that personalized outdoor activity interventions offered by volunteers may positively impact the quality of life of older adults, including tasks like errands and leisure activities. Additionally, some studies have focused on the integration and application of emerging technologies or remote services. Chen et al. [15] found that volunteers delivering home training courses using tablet devices could improve emotional well-being, cognitive abilities, and instrumental activities of daily living among older adults with cognitive impairments. Pichan et al. [16] demonstrated that during the COVID-19 pandemic, trained volunteers providing one-on-one telehealth support via telephone helped alleviate mental health issues, enhance community engagement, and reduce healthcare utilization among older adults.

Despite valuable contributions in areas such as resource coordination, service strategies, and technology integration, existing research still lacks sufficient focus on identifying community-dwelling older adults who are willing to receive volunteer services. This gap not only hinders the precise allocation of volunteer service resources but also results in inefficiencies and service blind spots, making it difficult for those most in need to receive effective support. To improve the efficiency of volunteer resource matching and accurately identify target service populations, it is essential to understand the key factors influencing older adults’ willingness to accept volunteer services. Although systematic studies specifically exploring the determinants of such willingness remain scarce, existing literature on unmet needs, healthcare demands, and long-term care requirements among older adults provides useful references for selecting relevant variables in this study. For example, Zhu [17] found that community-dwelling older adults who were younger, had poorer activities of daily living (ADL) capabilities, suffered from disabilities, displayed pessimistic emotions, had non-child primary caregivers, or anticipated greater opportunities for neighborhood mutual assistance were more likely to experience long-term unmet needs. Qin et al. [18] reported that older adults with disabilities who lived with their children, had stable income, and participated in health insurance programs showed significantly higher demand for long-term care services. While these studies do not directly focus on volunteer services, they offer important theoretical guidance for variable selection and model development in this research.

Based on this foundation, the study identifies target groups for volunteer services from two dimensions: (1) willingness to receive services, measured by a single binary item, and (2) intensity of service needs, assessed by a self-developed multi-item scale across various domains. By systematically analyzing this feedback, the study aims to identify individuals with both high willingness and high levels of need, thereby guiding volunteer efforts toward stratified and prioritized service delivery to the most vulnerable and urgently needing populations. Additionally, to enhance the accuracy and efficiency of target identification, the study incorporates multiple machine learning techniques [19, 20], including LASSO (Least Absolute Shrinkage and Selection Operator) for feature selection, decision trees, K-nearest neighbors, random forests, and support vector machines [21, 22]. By comparing these models in terms of variable relationship modeling, feature selection, noise reduction, and generalization performance [23]. Overall, this study aims to identify key factors influencing the willingness and demand levels for volunteer services among home-dwelling older adults and to analyze their importance, thereby providing a basis for service delivery and policy development.

## Method

### Design

This study employed an cross-sectional exploratory design. To address the complexity of accurately identifying individuals with genuine needs and willingness to receive help, the study was designed with a clear operational logic and data collection strategy, which were described as follows. In real life, older adults may underestimate or overstate their actual needs for various reasons. Some may exaggerate their needs to obtain additional resources, while others may conceal them due to distrust or fear of external intervention. These factors complicate the accurate identification of those who genuinely require assistance. Furthermore, even when older adults faced real difficulties in daily life, a lack of willingness to accept help could render volunteer efforts ineffective. To address these challenges, this study employed a structured questionnaire to collect older adults’ subjective feedback on both their willingness to receive help and their actual service needs. Compared with informal self-reporting, this structured approach offered greater consistency and reliability [24, 25]. Through systematic analysis of the responses, the study aimed to identify individuals with both high willingness and high levels of need, thereby enabling stratified and prioritized delivery of volunteer services to the most vulnerable and urgently needing populations.

It is important to note that this study specifically selected classification models such as Random Forest, Decision Tree, Support Vector Machine (SVM), and K-Nearest Neighbors (KNN) for comparative analysis, based on their respective advantages and adaptability to different data characteristics. Random Forest, which employs multiple decision trees and random feature selection, can reduce the risk of overfitting and handle complex linear relationships, and provides feature importance scores; however, its composite structure can be difficult to interpret [26]. Decision Trees handle both numerical and categorical data and offer intuitive visualization of the decision-making process, though they are prone to overfitting [27]. Support Vector Machines are particularly suitable for datasets with a large number of features, but the model is relatively difficult to interpret and its performance may be limited [28]. K-Nearest Neighbors is versatile and can process various types of data, but it faces challenges in selecting appropriate distance metrics and optimal K values [29]. Additionally, although other machine learning models, such as neural networks, perform well in certain domains, they typically require large training datasets. When data are limited, they are more prone to overfitting, and their decision-making processes are difficult to interpret. Therefore, neural networks were not included in this study.

### Participants

The study was conducted from March 25 to May 5, 2022, using a convenience sampling method. Older adults were recruited from four communities in four cities in central China (Wuhan, Ezhou, Zhengzhou, and Xinxiang). The selection of these sites was based on several considerations: alignment with National development strategies for central regions, geographic accessibility, feasibility of collaboration with local institutions, and the research team’s existing connections with community organizations. Inclusion criteria were: (1) aged 60 years or older, in accordance with the official definition of older adults in China as a developing country [30]; (2) currently living in an urban community in China for more than one year; (3) informed about the purpose and significance of the study and willing to participate. Exclusion criteria: individuals with communication barriers, such as hearing, speech, or cognitive impairments, were excluded because the data collection relied on structured self-report questionnaires that required participants to accurately understand and respond to the survey items

### Sample

To determine an appropriate sample size, the estimation formula for cross-sectional studies $n=\frac{Z_{1-\alpha/2}^2\times P\times\left(1-P\right)}{d^2}$ was used [31]. In this formula, n represents the sample size, Z is the critical value for a specific confidence level, P is the proportion of individuals expected to have a demand for volunteer services [31], and d is the allowable error. Assuming a 95% confidence interval (Z = 1.96), an expected demand proportion of 46.7% (14/30), and an allowable error of 0.05, the required sample size was calculated to be 423 participants, including a 10% non-response rate. To ensure an adequate sample size for logistic and multiple linear regression analyses, the calculation followed the principle that the sample size was at least 5–10 times the number of independent variables [32]. For logistic regression, considering the inclusion of 33 independent variables and an expected proportion of individuals with a demand for volunteer services, with a 10% non-response rate, the required sample size was at least 785 participants $\left(\frac{\left(33\times10\right)/46.7\%}{1-10\%}\right)$. For multiple linear regression, the same considerations applied, resulting in a required sample size of at least 785 participants $\left(\frac{33\times10}{\left(1-10\%\right)\times46.7\%}\right)$. In addition, for nonparametric models such as decision trees, random forests, K-nearest neighbors, and support vector machines, at least 500 observations are generally recommended to ensure model stability and generalizability [33]. In conclusion, the final required sample size was determined to be at least 785 participants.

### Instruments

The General Information Questionnaire was designed by the researchers to collect sociodemographic and current living conditions of older adults, including gender, age, education level, religious beliefs, previous occupation, marital status, type of medical insurance, whether living alone, whether without a caregiver, parental status, number of children, whether having a pension, financial support from relatives or friends, government subsidies, child support, whether the residence has an elevator, willingness for mutual assistance among older adults, ease of going out, health status, and a willingness to receive volunteer services.

The Volunteer Service Needs Assessment Scale for Community-Dwelling Older Adults was developed by the researchers, combined with qualitative research, literature review, and expert consultation, and included four dimensions: life assistance, health maintenance, visiting and communication, and social interaction, for a total of 27 items. Each item was scored on a 5-point Likert scale ranging from 1 (not needed at all) to 5 (very much needed). The total score ranged from 27 to 135, with higher scores indicating a greater overall demand for volunteer services. The Cronbach’s α of this scale was 0.959 in this study. The English language version of this questionnaire is provided in Appendix I.

The Perceived Caring Questionnaire for Community-Dwelling Older Adults was developed by the researchers under the guidance of social ecological system theory, combined with qualitative research, literature review, and expert consultation. It consisted of two parts: the first part concerned family caring perception, with 4 items including spouse care, care from cohabiting family members, care from non-cohabiting family members, and care from relatives; the second part concerned social caring perception, with 7 items including care from old friends, care from affiliated institutions, care from students or subordinates, care from neighbors, care from visited medical institutions, care from social volunteer services, and care from the government. Responses to family and social caring perception questions were “yes” or “no”. The Cronbach’s α of this scale was 0.730 in this study. The English language version of this questionnaire is provided in Appendix II.

The Activity of Daily Living Scale (ADL) was developed by Mahoney and Barthelin 1965 and is mainly used to assess the self-care ability of older adults or individuals with chronic illnesses in daily life [34]. The scale consists of 10 items, with different scoring ranges depending on the specific item. For example, some items are scored 0, 5, 10 points, while others are scored 0, 5, 10, 15 points. The total score ranges from 0 to 100, with higher scores indicating greater self-care ability in daily activities. The Cronbach’s α of this scale was 0.905 in this study.

The Patient Health Questionnaire (PHQ-9) was developed by Kroenke et al. based on DSM-IV criteria and is used for screening, diagnosing, and monitoring depression in adult patients [35]. The questionnaire consists of 9 items, each scored on a 4-point Likert scale from 0 (not at all) to 3 (nearly every day). The total score ranges from 0 to 27, with higher scores indicating more severe depression. The Cronbach’s α of this scale was 0.921 in this study.

### Data collection

Data were collected by the researchers and several other members of the research team, all of whom were graduate students in nursing. All researchers received standardized training to clarify the objectives and key points in this study. Before the survey began, the investigators contacted and obtained permission from community leaders or their respective administrative departments to ensure smooth data collection. Before conducting the official survey, the researchers carried out a pilot survey with 30 older adults in the community to refine language, identify potential issues and challenges during the survey, confirm the feasibility and validity of the research methods, and plan subsequent progress and resources accordingly. The pilot results also served as the basis for calculating the sample size. Subsequently, the researchers obtained contact and residential information for older residents from community management offices. To protect personal information, the researchers contacted eligible older adults or their family members individually by phone within the community management office. After confirming that participants met the inclusion and exclusion criteria and obtaining informed consent, community staff accompanied the researchers to the participants’ homes. Before distributing the questionnaire, the researchers reconfirmed that participants met the criteria and asked them to sign an informed consent form. Since all participants were over 60 years old, and some had limited education, the researchers neutrally read each item of the questionnaire. Some participants completed the paper-based questionnaire independently or used the WeChat app to scan a QR code to complete an online version. Researchers supervised the questionnaire completion process and provided necessary explanations to ensure data quality. After completing the survey, the researchers evaluated the questionnaires for correctness. After completing the survey, the researchers evaluated the questionnaires for completeness and correctness. If any responses were missing or unclear, the questionnaire was promptly returned to the participant for clarification and correction, with neutral guidance provided as needed. This procedure ensured that the final dataset contained no missing values and maintained high data quality. The survey followed principles of anonymity and confidentiality.

### Data analysis

Statistical analysis was conducted using SPSS 26.0, while Python was employed for advanced machine learning tasks. The significance level was set at 0.05.

### General data processing

Measurement data with normal distribution were presented as mean ± standard deviation (Mean ± SD) and were analyzed using t-tests or one-way ANOVA. Skewed data were expressed as median [M (P25, P75)] and were analyzed using non-parametric tests (e.g., Mann-Whitney U test). Categorical data were summarized using frequency (n) and percentage (%), and were analyzed using chi-square tests (χ² test).

#### Phase I: factors influencing willingness to receive volunteer services

A univariate analysis was conducted first, followed by binary logistic regression. The dependent variable was willingness (yes/no), and independent variables were those significant in univariate analysis (*P* < 0.05), further screened using LASSO (Least Absolute Shrinkage and Selection Operator). Although no formal distinction between independent variables and covariates was made, all selected variables were treated as independent variables based on their potential explanatory power. To evaluate model fit, the Hosmer-Lemeshow test and multicollinearity diagnostics (tolerance and VIF) were conducted. Additionally, four classification models—logistic regression, random forest (RF), decision tree (DT), support vector machine (SVM), and K-nearest neighbors (KNN)—were trained using Python. Model performance was assessed using the Receiver Operating Characteristic (ROC) curves and Area Under the Curve (AUC), with Delong tests for pairwise comparisons [36]. To develop and validate machine learning models, the dataset was randomly split into a training set (70%) and a test set (30%). This 70/30 split is commonly adopted in machine learning research to ensure sufficient data for both model training and performance evaluation [37].

#### Phase II: factors influencing demand level for volunteer services

A univariate analysis was conducted with the demand score as the outcome, followed by multiple linear regression using LASSO-filtered predictors. The Kolmogorov–Smirnov test, Q-Q plots, and residual scatter plots were used to assess normality and homoscedasticity. Multicollinearity was tested via tolerance and VIF, and independence of residuals was verified using the Durbin-Watson statistic. Model performance was validated using mean absolute error (MAE), mean squared error (MSE), root mean squared error (RMSE), and R².

#### Phase III: ranking of influencing factors

To compare the relative importance of predictors across models: For regression models, both the absolute value of the unstandardized regression coefficient (B) and standardized coefficient (β) were considered. Since SPSS does not directly provide β in logistic regression, β was estimated manually using the formula: $b'j=b_jS_j/\left(\pi/\sqrt3\right)$ [38]. For classification models, variable importance was ranked based on feature importance scores from the random forest algorithm.

## Results

A total of 946 questionnaires were distributed, with 852 returned, resulting in a response rate of 90.1%. After excluding invalid questionnaires, 828 valid responses were included, yielding an effective response rate of 87.5%. The included 828 older adults ranged in age from 60 to 97 years, with a median age of 69 (65, 76) years. Among them, 66.2% had attained at least a junior high school education, and 88.9% reported no religious affiliation. In terms of self-rated health, 54.7% considered their health to be “very good” or “good.” Most participants (87.2%) did not live alone, and 42.1% reported having no caregiver. Approximately 69.7% expressed a willingness to engage in mutual aid. Regarding family circumstances, 75.4% had two or more children, and 77.4% had lost both parents. Financial support from children was reported by 39.5% of participants. A majority (77.8%) reported ease of going out, and 74.3% were married. Concerning social security, 41.7% were enrolled in urban employee medical insurance, and 71.0% received a pension. Only 10.1% received government subsidies. Additionally, 35.9% lived in buildings with elevators, and 85.6% received financial support from relatives or friends. Detailed information is presented in Table 1.

**Table 1:** General information of community-dwelling older adults

Variable	*N* (%)
Age (years)	
60∼69	424 (51.2)
70∼79	268 (32.4)
≥ 80	136 (16.4)
Gender	
Male	365 (44.1)
Female	463 (55.9)
Education level	
Primary school education or under	280 (33.8)
Junior high school education	250 (30.2)
Senior high school education or above	298 (36.0)
Religious beliefs	
Yes	92 (11.1)
No	736 (88.9)
Previous occupation	
Civil servant	153 (18.5)
Worker	238 (28.7)
Farmer	202 (24.4)
Other	235 (28.4)
Health status	
Very good	119 (14.4)
Good	334 (40.3)
Average	301 (36.4)
Poor	68 (8.2)
Very poor	6 (0.7)
Living alone	
Yes	106 (12.8)
No	722 (87.2)
No caregiver	
Yes	349 (42.1)
No	479 (57.9)
Willingness for mutual aid	
Yes	577 (69.7)
No	251 (30.3)
Number of children	
≤ 1	204 (24.6)
2	315 (38.1)
≥ 3	309 (37.3)
Parents’ situation	
All alive	86 (10.4)
A single parent died	101 (12.2)
Both died	641 (77.4)
Child subsidies	
Yes	327 (39.5)
No	501 (60.5)
Convenience for going out	
Yes	644 (77.8)
No	184 (22.2)
Marital status	
Married	615 (74.3)
Other*	213 (25.7)
Type of medical insurance	
Urban employees insurance	345 (41.7)
Urban residents insurance	182 (22.0)
New rural cooperative medical insurance	205 (24.8)
Other insurance	96 (11.6)
Pension	
Yes	588 (71.0)
No	240 (29.0)
Government subsidies	
Yes	84 (10.1)
No	744 (89.9)
Elevator in residence	
Yes	297 (35.9)
No	531 (64.1)
Financial support from relatives/friends	
Yes	709 (85.6)
No	119 (14.4)

“Other” refers to individuals who are unmarried, divorced, or widowed

### Phase I: willingness to receive volunteer services

Univariate analysis identified 24 variables with statistically significant differences (Appendix III). Further feature selection using LASSO regression identified 17 key predictors. The dataset was split into a training set (70%, 580 samples) and a test set (30%, 248 samples). Five classification models—logistic regression, random forest, decision tree, support vector machine (SVM), and K-nearest neighbors (KNN)—were trained using the training set, and their performance was evaluated using the test set.

**Fig. 1:**
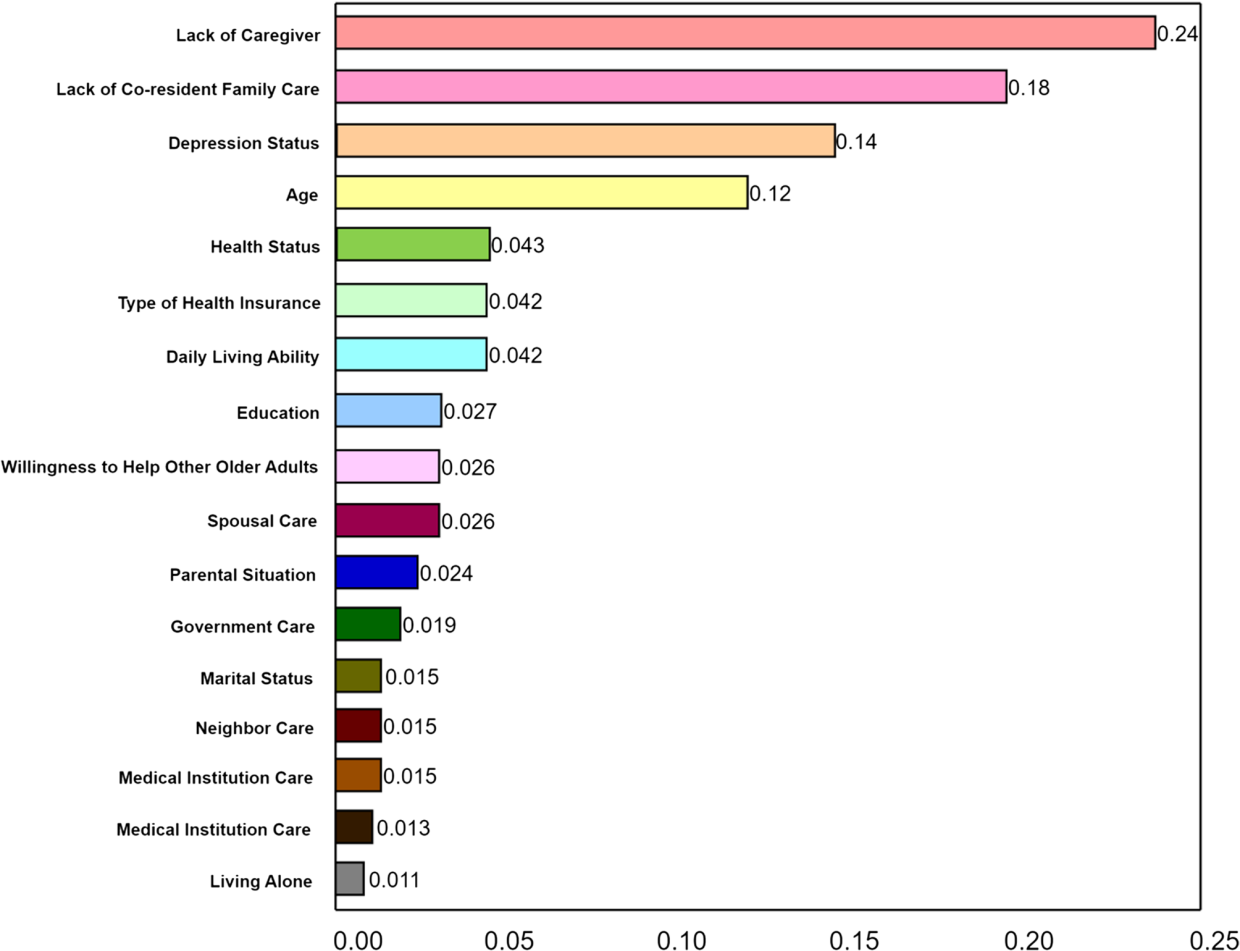
Bar chart of feature importance for random forest

Comparing ROC curves and AUC values, logistic regression and random forest showed high AUC values (0.975 and 0.970, respectively), followed by decision tree (0.879) and KNN (0.878). SVM had the lowest AUC value (0.833) (Fig. 1). Core metrics such as accuracy, F1 score, precision, recall, specificity, and youden’s index are presented in appendix IV. The Delong test showed no significant difference between logistic regression and random forest (*P* = 0.746), while both outperformed the other three models (*P* < 0.001, appendix V). Thus, logistic regression and random forest demonstrated superior performance.

**Fig. 2:**
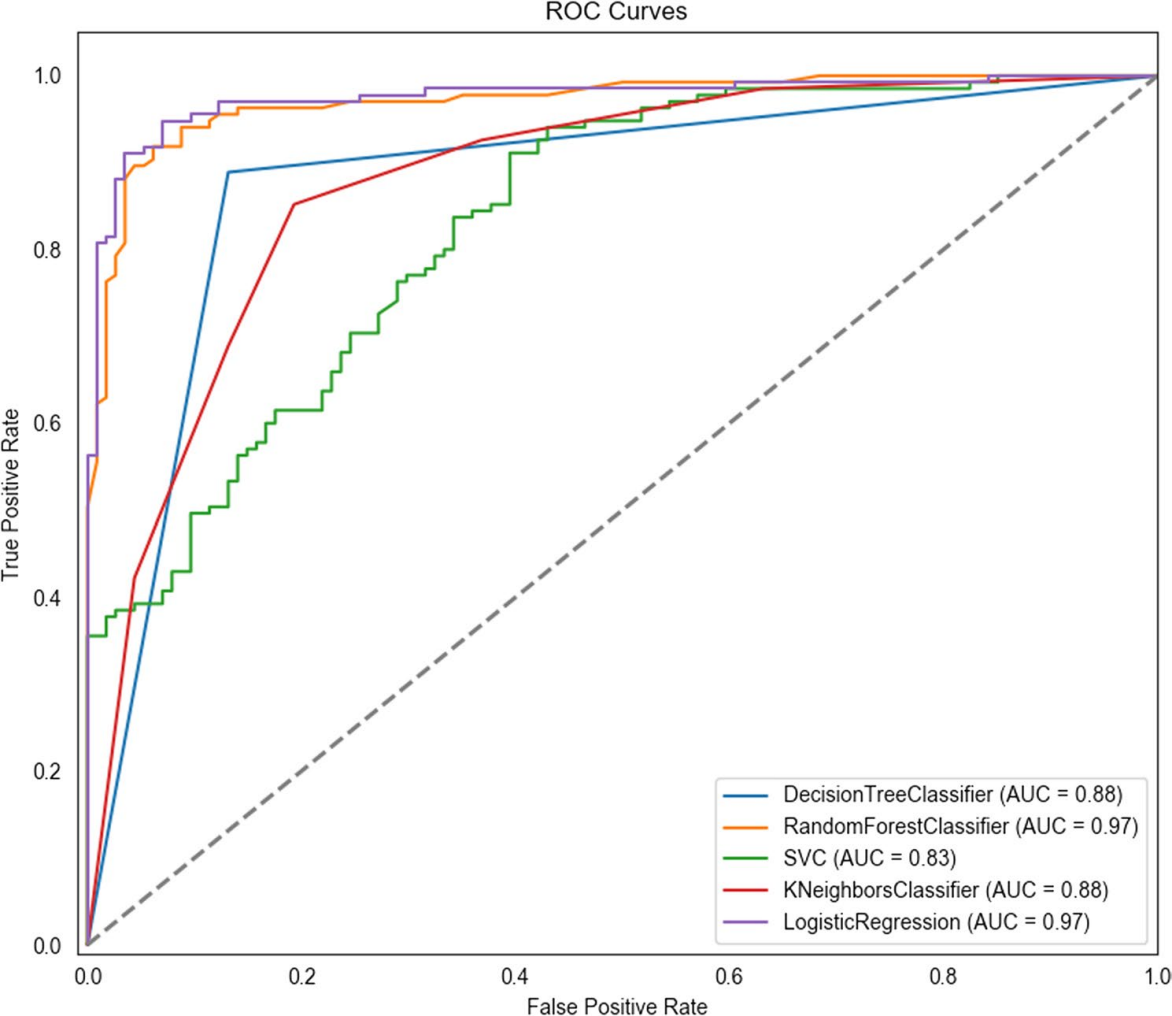
Comparison of ROC curves for different prediction models

In binary logistic regression analysis, the Hosmer-Lemeshow test indicated a good model fit (*P* = 0.817 > 0.05). Multicollinearity diagnostics showed tolerance values between 0.445 and 0.911 (all > 0.1) and variance inflation factors (VIFs) between 1.097 and 2.247 (all < 10), indicating no multicollinearity issues. Logistic regression analysis identified independent variables including age, health status, living alone, lack of caregiving, depression status, activities of daily living, cohabiting family care, type of medical insurance, neighbor care, government care, and willingness for mutual assistance as independent influencing factors for receiving from volunteer services among older adults (Table 2). In the random forest model, the data were split into a 70% training set and a 30% test set, with a random seed of 100 for reproducibility, using 100 decision trees. Figure 2 showed the variable importance ranking based on LASSO regression analysis.

**Table 2:** Binary logistic regression analysis of the willingness to receive volunteer services (*n* = 828)

Variable	OR	95%CI	*P*
Age (years)	1.115	[1.067, 1.164]	<0.001
Health status	1.625	[1.113, 2.374]	0.012
Living alone	0.068	[0.019, 0.238]	<0.001
No caregiver	45.493	[21.118, 98.001]	<0.001
Willingness for mutual aid	3.981	[2.064, 7.678]	<0.001
Care from co-habiting family	0.013	[0.004, 0.036]	<0.001
Care from neighbors	0.454	[0.226, 0.912]	0.027
Care from government	4.483	[2.268, 8.861]	<0.001
Depression (score)	1.267	[1.179, 1.362]	<0.001
Daily living ability (score)	1.028	[1.008, 1.049]	0.006
Type of medical insurance (Reference Group = Other)			
Urban employee insurance	7.752	[2.792, 21.523]	<0.001
Urban resident insurance	3.845	[1.263, 11.712]	0.018
New rural cooperative health insurance	4.624	[1.546, 13.826]	0.006

### Phase II: demand level for volunteer services

Univariate analysis identified 15 variables with significant differences (Appendix VI), and LASSO regression narrowed these to nine key predictors. The dataset was split into a training set (70%, 330 samples) and a test set (30%, 142 samples), and the models were trained using the three regression methods: multiple linear regression, random forest regression, and support vector regression. Model performance was validated using key metrics including Mean Absolute Error (MAE), Mean Squared Error (MSE), Root Mean Squared Error (RMSE), and R-squared (R²), as shown in Appendix VII. Results indicated that the multiple linear regression model performed best compared to the other two models.

The Kolmogorov-Smirnov test, along with histogram and Q-Q plot analysis, confirmed normal distribution of residuals. The standardized residual scatter plot showed no increase in variance as predicted values increased, supporting the assumption of homoscedasticity. Multicollinearity diagnostics showed tolerance values between 0.916 and 1.000 (all > 0.1) and variance inflation factors (VIFs) between 1.000 and 1.091 (all < 10), indicating no multicollinearity issues. The Durbin-Watson statistic of 1.737 confirmed the independence of residuals. Multiple linear regression analysis with the demand for volunteer services as the dependent variable identified independent variables including depression status, health status, willingness for mutual assistance, and government care as independent influencing factors (Table 3).

**Table 3:** Multiple linear regression analysis of volunteer service demand (*n* = 472)

Variable	B	SE	β	t	*P*	95%CI
Constant	74.473	3.567		20.879	<0.001	[67.464, 81.482]
Depression status	1.981	0.161	0.498	12.341	<0.001	[1.666, 2.296]
Health status	−3.247	1.033	−0.129	−3.144	0.002	[−5.276, −1.217]
Willingness for mutual aid	5.719	2.010	0.115	2.846	0.005	[1.770, 9.668]
Care from government	4.752	1.820	0.103	2.611	0.009	[1.175, 8.328]

B = unstandardized coefficient; SE = standard error; β = standardized coefficient calculated to reflect the relative importance of each predictor

### Phase III: comprehensive importance ranking

To determine the importance ranking of factors influencing whether community-dwelling older adults have a willingness to receive volunteer services and the extent of that demand, this study conducted a comprehensive analysis based on multiple regression and machine learning methods.

For the presence of the willingness to receive volunteer service, the importance ranking of influencing factors was derived based on binary logistic regression results, considering both β values and t-values (see Table 4). In combination with random forest model analysis, the importance ranking of influencing factors was determined. High importance factors included those ranked in the top five in both logistic regression and random forest analyses: absence of cohabiting family care and lack of caregiving. Medium importance factors included those ranked in the top five in either analysis: living alone, having basic medical insurance, higher levels of depression status, older age, and poor health status. Low importance factors included other key predictors: willingness to help other older adults, government care, lack of neighbor care, independent activities of daily living, primary school education or below, lack of spouse care, both parents deceased, marital status, absence of care from medical institutions, and lack of employer care.

**Table 4:** Importance ranking of influencing factors based on logistic regression

Rank	Variable	B	SE	β
1	Care from co-habiting family	4.376	0.532	1.284
2	Living alone	2.688	0.638	0.945
3	No caregiver	−3.818	0.392	0.825
4	Urban employee health insurance	2.048	0.521	0.588
5	New rural cooperative health insurance	1.531	0.559	0.472
6	Urban resident health insurance	1.347	0.568	0.422
7	Government care	−1.500	0.348	0.288
8	Willingness for mutual aid	−1.381	0.335	0.255
9	Care from neighbors	0.789	0.356	0.155
10	Health status	0.486	0.193	0.052
11	Depression status	0.237	0.037	0.005
12	Age (years)	0.108	0.022	0.001
13	Daily living ability (score)	0.028	0.010	0.000

B = unstandardized coefficient; SE = standard error; β = standardized coefficient calculated to reflect the relative importance of each predictor

**Fig. 3:**
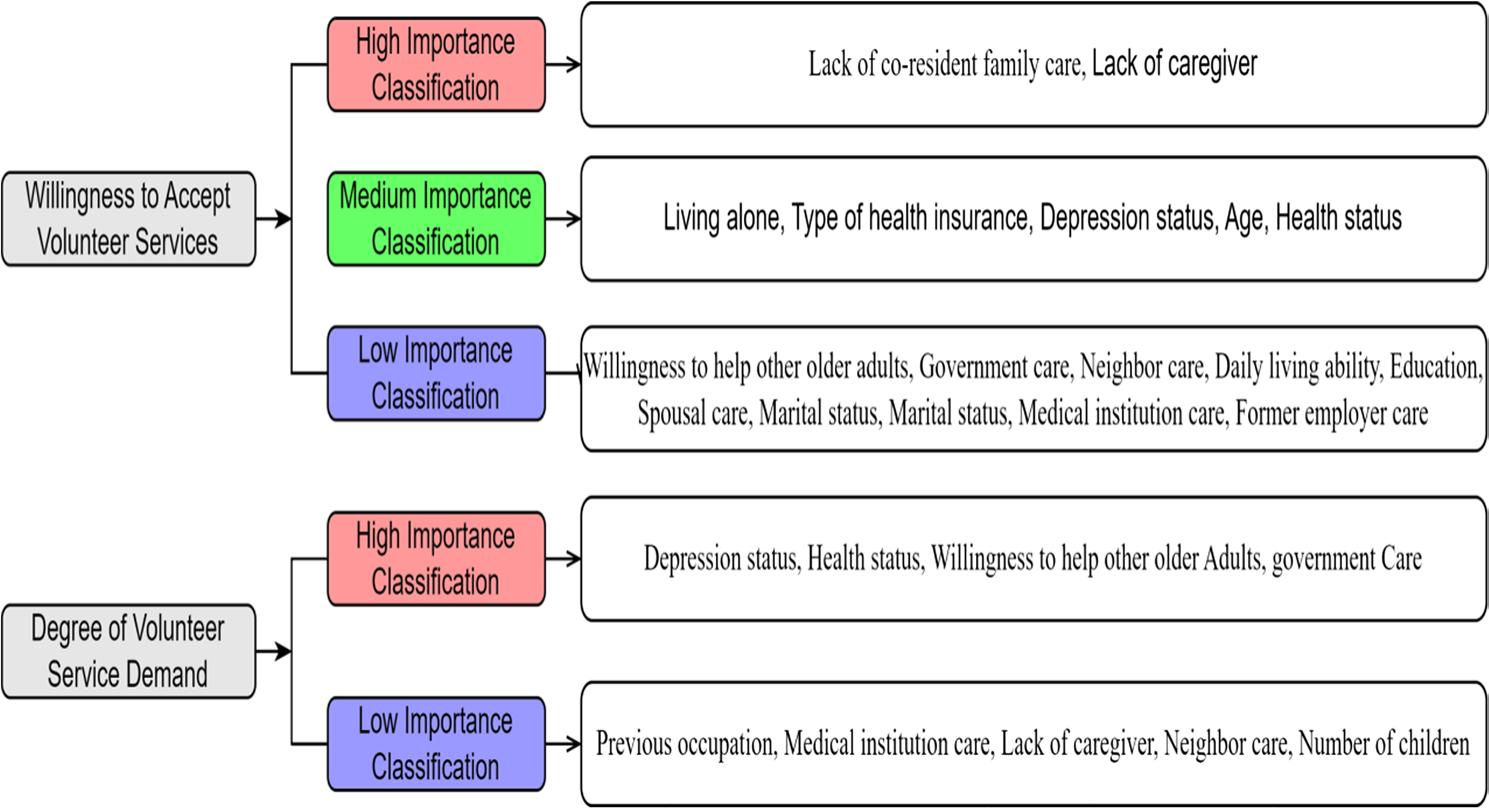
Importance classification of influencing factors

For the extent of volunteer service demand, the importance ranking of influencing factors was based on multiple linear regression results, considering both β values and t-values. High importance factors included those with significant influence: depression status, poor health status, willingness to help others, and government care. Low importance factors included other predictors: being a farmer, lack of care from medical institutions, availability of caregiving, neighbor care, and having two or more children. Drawing on the comprehensive factor importance analysis, this study outlined potential characteristics of community-dwelling older adults who may have strong willingness to receive volunteer services (Fig. 3).

### Summary of main findings

This study identified key factors influencing both the willingness and demand level for volunteer services among community-dwelling older adults. The best-performing models for predicting willingness were logistic regression and random forest, both demonstrating high accuracy. The most critical predictors of willingness included the absence of cohabiting family care, lack of caregiving support, living alone, poor health and depression status, having basic medical insurance, and advanced age. For demand level, multiple linear regression yielded the best performance, with depression, poor health, willingness to help others, and lack of government care emerging as significant factors. These findings offer valuable insights for the targeted design and delivery of volunteer support programs.

## Discussion

This study identified multiple variables significantly associated with both the willingness to receive volunteer services and the degree of service demand through univariate analysis. Given the large number of significant variables, which could hinder the focus on core influencing factors, the LASSO method was employed to optimize variable selection, simplify the model, and enhance generalizability [39, 40]. However, even after LASSO processing, a considerable number of potential predictors remained. Therefore, several classification models, including logistic regression, random forest, decision tree, support vector machine (SVM), and k-nearest neighbors (KNN), were constructed and compared to select the most interpretable and practically applicable model.

During model selection, the DeLong test and key performance comparisons revealed that logistic regression and random forest performed best for predicting willingness, whereas multiple linear regression performed best for assessing demand. Each model has unique strengths: for instance, random forest effectively handles complex relationships and provides variable importance scores, while decision trees offer clear visual decision pathways. However, some models have interpretability limitations, such as random forest and SVM, which exhibit “black-box” characteristics [26, 28]. This lack of transparency may hinder their practical utility in fields like social sciences and health services, where clear reasoning behind predictions is often essential for decision-making. Overall, the combined use of LASSO regression and multi-model comparison enhanced the scientific rigor and accuracy of model selection in this study. This interdisciplinary approach provides a scientific and effective analytical framework for future research in similar fields, particularly those involving datasets with numerous significant variables and complex modeling challenges.

Given the numerous important factors identified, this study focuses on the most significant variables related to the two dependent outcomes for further in-depth exploration and analysis. Firstly, regarding the willingness to receive volunteer services, the absence of cohabiting family members’ care and the lack of caregivers were identified as the most influential factors. This finding is supported by several previous studies [17, 18]. One study indicated that older adults whose primary caregivers were non-family members or lacked caregiving willingness faced a significantly increased risk of unmet long-term care needs [17]. Another study found that disabled older adults not living with their children had significantly higher demand for integrated medical and nursing services, suggesting that the presence and care of family members greatly influence their willingness to accept such services [18]. Older adults without cohabiting family members or caregivers are at an increased risk of experiencing social isolation and mental health challenges [41], as they lack reliable caregiving support and daily family assistance. Consequently, the difficulties they encounter are often left unaddressed. Therefore, interventions aimed at supporting this group should prioritize the establishment and reinforcement of community-based support networks. Recommended strategies include fostering long-term relationships with volunteers, enhancing opportunities for social engagement, and promoting mutual assistance within communities and neighborhoods [42, 43]. In addition, providing personalized services such as home visits, psychological counseling, and health management should be incorporated as complementary interventions [44].

Secondly, in terms of the degree of demand for volunteer services, depressive tendencies, poor health status, willingness to assist other older adults, and receipt of government care were identified as the most significant predictors of demand. Older adults with depressive tendencies tend to exhibit a higher demand for volunteer services, likely due to their increased concerns about health and social functioning. This finding is consistent with research by Wu Shaofeng [45], which indicates that depressive conditions in urban community-dwelling older adults significantly predict their need for life care, healthcare, and mental comfort. The severity of depression in older adults is a critical concern, as it can be associated with conditions such as dementia or even suicidal ideation [46, 47]. It is noteworthy that volunteer home visits have been shown to substantially reduce negative emotional states in older adults, particularly mental distress and suicidal ideation in women [48, 49]. Furthermore, older adults with poor health not only have a heightened need for medical services but also experience psychological challenges due to prolonged physical discomfort, which hinders their social engagement and leads to increasingly complex and multifaceted needs. This observation aligns with the findings reported by Zou [50]. Therefore, it is particularly essential to encourage volunteers, especially those with a nursing background, to provide timely, targeted support to older adults experiencing depressive symptoms and poor health status. Such volunteers differ from professional nurses who provide clinical medical services or family caregivers responsible for daily care. They typically possess basic nursing knowledge and health awareness, enabling them to play a complementary and supportive role in the community by helping to identify potential health issues among older adults and offering humanistic care and preventive services [12, 51].

The results also show that older adults who receive government care generally exhibit higher demand for volunteer services. This reflects the fact that older adults under government care often face significant life difficulties, which attract governmental attention. Moreover, after receiving external support and experiencing tangible benefits, this group may become more proactive in seeking further support to meet their practical needs. As a result, volunteers with a nursing background should place greater emphasis on this group and adopt targeted service measures. Additionally, the demand for volunteer services is higher among older adults who are willing to help others. This is likely because older adults who are willing to help others typically have a strong sense of social participation, which encourages them to actively express their own needs [52]. Furthermore, in the process of helping others, they are more likely to identify various problems and, thus, urgently need broader support to address these challenges. This aligns with our findings that willingness to help others is a significant predictor of higher service demand. Therefore, volunteers should not ignore the needs of this group of older adults but should actively offer support, which may further stimulate their willingness to help others, creating a virtuous cycle that benefits a broader group of older adults. Recent research indicates that older adults living in areas with high volunteer group participation have a lower risk of depression, regardless of whether they participate in volunteer activities themselves [53]. This finding further highlights the importance of supporting older adults who are willing to help others. In addition to the high-importance factors identified, the important influencing factors revealed in the middle and low-importance categories also provide valuable insights for volunteers to determine priority service targets. These factors primarily include the personal characteristics, health status, and social support situation of older adults. These variables offer practical value in refining service stratification when resources are limited.

The findings of this study provide strong support for addressing issues of resource mismatch, inefficiency, and missed service opportunities in community-based volunteer services. By identifying key factors influencing older adults’ willingness to receive volunteer services and the intensity of their needs, the study enables precise classification and stratification of service recipients, prioritizing vulnerable groups with high needs and strong willingness. The use of LASSO regression and multi-model comparisons helps optimize variable selection and reduces waste and blind spots. Moreover, the study highlights high-risk groups such as those lacking caregivers or exhibiting depressive tendencies, offering clear direction for targeted volunteer interventions. Overall, this study provides both theoretical and empirical support for building an efficient, precise, and responsive volunteer service system.

### Limitation

#### Study design and sample coverage

this study employed a cross-sectional design, which limits the ability to track changes over time and establish causal relationships. Future research should consider using a longitudinal design and probability-based sampling methods to enhance sample representativeness and explore the long-term effects of influencing factors. Moreover, the data were collected from relatively developed cities in central China and did not include rural areas, towns, or less-developed regions, limiting generalizability.

#### Participant selection and variable scope

To ensure reliable data, older adults with communication barriers were excluded. Although this approach helped maintain data quality and contributed to a high response rate—supported by face-to-face data collection, trained interviewers, and community staff—it may have introduced selection bias. Both individuals with communication difficulties and those who declined participation could represent vulnerable groups with higher unmet needs, potentially leading to an underestimation of overall service demand. In addition, the study primarily focused on family- and social-level factors, leading to the exclusion of certain individual-level health-related variables, such as detailed medical conditions and polypharmacy records.

#### Responsiveness to immediate needs

The study design did not address urgent or immediate needs in a timely manner. Therefore, while the current findings provide useful insights, incorporating feedback and suggestions from stakeholders such as the community and neighbors is also important to more accurately identify target groups for volunteer services.

## Conclusion

This study employed LASSO regression and multiple machine learning models to identify the potential key characteristics of community-dwelling older adults with a strong willingness to receive volunteer services. The best-performing models for predicting willingness were logistic regression and random forest, both demonstrating high accuracy. The most critical predictors of willingness included the absence of cohabiting family care, lack of caregiving support, living alone, poor health and depression status, having basic medical insurance, and advanced age. For demand level, multiple linear regression yielded the best performance, with depression, poor health, willingness to help others, and lack of government care emerging as significant factors. These findings provide a basis for targeted design and delivery of volunteer support programs and support further optimization of volunteer resource allocation and stratified service strategies.

## Supplementary Information

Supplementary Material 1: Appendix I. English version of this Volunteer Service Needs Assessment Scale. Appendix II. English version of Perceived Caring Questionnaire. Appendix III. Comparison of influencing factors between older adults willing and unwilling to receive volunteer services Appendix IV. Classification model performance evaluation based on different algorithms. Appendix V. Differences in model performance using delong test. Appendix VI. Impact of influencing factors on older Individuals’ demand for volunteer services. Appendix VII. Evaluation of Regression Model Performance Based on Different Algorithms.

## Data Availability

I declare that all data and materials are available from the corresponding author upon reasonable request.
