# Supplementary material for: Identifying vulnerable groups of community-dwelling older adults with a strong willingness to receive volunteer services based on machine learning methods
Source: BMC Public Health. 2025 Nov 18;25:4033. doi: 10.1186/s12889-025-24902-7 (PMC12625264; doi:10.1186/s12889-025-24902-7)
Supplement: Supplementary file 1 — Supplementary Material 1: Appendix I. English version of this Volunteer Service Needs Assessment Scale. Appendix II. English version of Perceived Caring Questionnaire. Appendix III. Comparison of influencing factors between older adults willing and unwilling to receive volunteer services Appendix IV. Classification model performance evaluation based on different algorithms. Appendix V. Differences in model performance using delong test. Appendix VI. Impact of influencing factors on older Individuals’ demand for volunteer services. Appendix VII. Evaluation of Regression Model Performance Based on Different Algorithms. [file 12889_2025_24902_MOESM1_ESM.docx]

Appendix I.

English version of this Volunteer Service Needs Assessment Scale for Community-Dwelling Older Adults.

| Q1 | Obtain assistance in purchasing groceries or shopping from voluntary services. |
| --- | --- |
| Q2 | Obtain assistance in delivering meals or cooking from voluntary services. |
| Q3 | Obtain assistance in maintaining personal hygiene from voluntary services. |
| Q4 | Obtain assistance in activities within the room from voluntary services. |
| Q5 | Obtain assistance in going out for activities or walks from voluntary services. |
| Q6 | Obtain assistance in cleaning from voluntary services. |
| Q7 | Obtain guidance on how to use mobile phones or the internet from voluntary services. |
| Q8 | Obtain assistance in handling deposits, withdrawals, or wealth management at the bank from voluntary services. |
| Q9 | Obtain assistance in measuring blood pressure, blood sugar, or conducting other physical examinations from voluntary services. |
| Q10 | Obtain medication instruction from voluntary services. |
| Q11 | Obtain guidance on health care and disease prevention from voluntary services. |
| Q12 | Obtain guidance on fall prevention from voluntary services. |
| Q13 | Obtain accompany for medical treatment or physical examination from voluntary services. |
| Q14 | Obtain timely response when seeking sought for help from voluntary services. |
| Q15 | Obtain guidance on physical rehabilitation and functional exercise from voluntary services. |
| Q16 | Be accompanied in chatting by volunteers. |
| Q17 | Obtain encouragement on speaking up and respond from voluntary services. |
| Q18 | Be celebrated for birthdays or holidays by volunteers. |
| Q19 | Be identified the negative emotions and obtain suggestions by volunteers. |
| Q20 | Be respected and appreciated by volunteers. |
| Q21 | Obtain strategies to address the concerns about the families from voluntary services. |
| Q22 | Obtain assistance in registering or contacting for social activities from voluntary services. |
| Q23 | Obtain assistance in participate in social activities from voluntary services. |
| Q24 | Obtain assistance in organize social activities from voluntary services. |
| Q25 | Obtain assistance in meeting more friends, clubs, or organizations from voluntary services. |
| Q26 | Be accompanied by volunteers in doing things they are interested in. |
| Q27 | Obtain a platform to showcase or cultivate one's interests and hobbies from voluntary services. |

The subject of all questions is 'home-based older adults’.

Appendix II.

English version of Perceived Caring Questionnaire for Community-Dwelling Older Adults.

| **Dimensions** | **Variables** | **Specific items** |
| --- | --- | --- |
| Family care | From elderly wives | I have received care from elderly wives. |
|  | From families of other cohabitants | I have received care from families of other cohabitants. |
|  | From families of non-cohabitants | I have received care from families of non-cohabitants. |
|  | From relatives | I have received care from relatives. |
| Social care | From government | I have received care from government departments. |
|  | From former affiliated institutions | I have received care from former work units. |
|  | From old friends | I have received care from old friends. |
|  | From subordinates or students | I have received care from subordinates or students. |
|  | From neighbors | I have received care from neighbors. |
|  | From visited medical institutions | I have received care from visited medical institutions. |
|  | From social voluntary services | I have received care from social voluntary services. |

Appendix III.

Comparison of influencing factors between older adults willing and unwilling to receive volunteer services (n=828)

| **Variable** | **Willingness to receive voluntary service** | | **Statistic Value** | ***P*** |
| --- | --- | --- | --- | --- |
|  | Yes | No |  |  |
| **Age (years), M (P25, P75)** | 72（66，79） | 66（64，71） | -9.195^b^ | ＜0.001 |
| **Education Level** |  |  | 10.039^a^ | 0.007 |
| Primary school education or under | 179（63.9） | 101（36.1） |  |  |
| Junior high school education | 126（50.4） | 124（49.6） |  |  |
| Senior high school education or above | 167（56.0） | 131（44.0） |  |  |
| **Previous Occupation** |  |  | 11.875^a^ | 0.008 |
| Civil servant | 92（60.1） | 61（39.9） |  |  |
| Worker | 147（61.8） | 91（38.2） |  |  |
| Farmer | 121（59.9） | 81（40.1） |  |  |
| Other | 112（47.7） | 123（52.3） |  |  |
| **Health Status** |  |  | 50.826^a^ | ＜0.001 |
| Very good | 58（48.7） | 61（51.3） |  |  |
| Good | 150（44.9） | 184（55.1） |  |  |
| Fair | 211（70.1） | 90（29.9） |  |  |
| Poor | 49（72.1） | 19（27.9） |  |  |
| Very poor | 4（66.7） | 2（33.3） |  |  |
| **Willingness for Mutual Aid** |  |  | 8.494^a^ | 0.004 |
| Yes | 348（60.3） | 229（39.7） |  |  |
| No | 124（49.4） | 127（50.6） |  |  |
| **Depression status (score), M (P25, P75)** | 8（4，10） | 3（0，6） | -10.849^b^ | ＜0.001 |
| **Daily Living Ability (score), M (P25, P75)** | 100（100，100） | 100（97.5，100） | -5.390^b^ | ＜0.001 |
| **Marital Status** |  |  | 22.565 | ＜0.001 |
| Married | 321（52.2） | 294（47.8） |  |  |
| Other* | 151（70.9） | 62（29.1） |  |  |
| **Parents' Status** |  |  | 16.071 | ＜0.001 |
| All alive | 43（50.0） | 43（50.0） |  |  |
| A single parent died | 41（40.6） | 60（59.4） |  |  |
| Both died | 388（60.5） | 253（39.5） |  |  |
| **Living Alone** |  |  | 22.496 | ＜0.001 |
| Yes | 83（78.3） | 23（21.7） |  |  |
| No | 389（53.9） | 333（46.1） |  |  |
| **No Caregiver** |  |  | 296.135 | ＜0.001 |
| Yes | 320（91.7） | 29（8.3） |  |  |
| No | 152（31.7） | 327（68.3） |  |  |
| **Care from Spouse** |  |  | 57.031 | ＜0.001 |
| Yes | 310（49.6） | 315（50.4） |  |  |
| No | 162（79.8） | 41（20.2） |  |  |
| **Care from Co-habiting Family** |  |  | 268.297 | ＜0.001 |
| Yes | 204（37.0） | 347（63.0） |  |  |
| No | 268（96.8） | 9（3.2） |  |  |
| **Care from Non-cohabiting Family** |  |  | 3.863 | 0.049 |
| Yes | 440（56.2） | 343（43.8） |  |  |
| No | 32（71.1） | 13（28.9） |  |  |
| **Care from Relatives** |  |  | 31.762 | ＜0.001 |
| Yes | 376（53.0） | 333（47.0） |  |  |
| No | 96（80.7） | 23（19.3） |  |  |
| **Care from Students or Subordinates** |  |  | 34.524 | ＜0.001 |
| Yes | 127（43.3） | 166（56.7） |  |  |
| No | 345（64.5） | 190（35.5） |  |  |
| **Care from Original Unit** |  |  | 29.811 | ＜0.001 |
| Yes | 155（45.7） | 184（54.3） |  |  |
| No | 317（64.8） | 172（35.2） |  |  |
| **Care from Neighbors** |  |  | 5.999 | 0.014 |
| Yes | 355（54.8） | 293（45.2） |  |  |
| No | 117（65.0） | 63（35.0） |  |  |
| **Child Subsidy** |  |  | 4.279 | 0.039 |
| Yes | 172（52.6） | 155（47.4） |  |  |
| No | 300（59.9） | 201（40.1） |  |  |
| **Type of medical insurance** |  |  | 49.693 | ＜0.001 |
| Urban employees insurance | 231（67.0） | 114（33.0） |  |  |
| Urban residents insurance | 104（57.1） | 78（42.9） |  |  |
| New rural cooperative medical insurance | 111（54.1） | 94（45.9） |  |  |
| Other insurance | 26（27.1） | 70（72.9） |  |  |
| **Retirement Pension** |  |  | 8.472 | 0.004 |
| Yes | 354（60.2） | 234（39.8） |  |  |
| No | 118（49.2） | 122（50.8） |  |  |
| **Care from Medical Institutions** |  |  | 12.647 | ＜0.001 |
| Yes | 201（50.6） | 196（49.4） |  |  |
| No | 271（62.9） | 160（37.1） |  |  |
| **Care from Volunteer Services** |  |  | 5.106 | 0.024 |
| Yes | 120（50.8） | 116（49.2） |  |  |
| No | 352（59.5） | 240（40.5） |  |  |
| **Care from Government** |  |  | 13.541 | ＜0.001 |
| Yes | 314（62.1） | 192（37.9） |  |  |
| No | 158（49.1） | 164（50.9） |  |  |

Note: ^a^ represents t-value. ^b^ represents F-value.

Appendix IV.

Classification model performance evaluation based on different algorithms (n=248)

| **Model** | **Accuracy** | **F1 Score** | **Precision** | **Recall** | **Specificity** | **Youden’s Index** | **AUC** |
| --- | --- | --- | --- | --- | --- | --- | --- |
| Logistic Regression | 0.928 | 0.927 | 0.927 | 0.929 | 0.939 | 0.867 | 0.975 |
| Random Forest | 0.916 | 0.914 | 0.917 | 0.913 | 0.886 | 0.799 | 0.970 |
| Decision Tree | 0.880 | 0.879 | 0.879 | 0.879 | 0.868 | 0.747 | 0.879 |
| Support Vector Machine | 0.747 | 0.739 | 0.754 | 0.737 | 0.623 | 0.360 | 0.833 |
| K-Nearest Neighbors | 0.831 | 0.830 | 0.830 | 0.829 | 0.807 | 0.636 | 0.878 |

Appendix V.

Differences in model performance using delong test

| **Model (P-value)** | **Logistic Regression** | **Random Forest** | **Decision Tree** | **Support Vector Machine** | **K-Nearest Neighbors** |
| --- | --- | --- | --- | --- | --- |
| Logistic Regression | - | - | - | - | - |
| Random Forest | 0.746 | - | - | - | - |
| Decision Tree | ＜0.001* | ＜0.001* | - | - | - |
| Support Vector Machine | ＜0.001* | ＜0.001* | 0.146 | - | - |
| K-Nearest Neighbors | ＜0.001* | ＜0.001* | 0.993 | 0.148 | - |

Note: * indicates statistical significance with P＜0.05.

Appendix VI.

Impact of influencing factors on older Individuals’ demand for volunteer services (n=472)

| **Variable** | | | | **n（%）** | | **Mean ± SD** | **Statistic Value** | **P** | | | |
| --- | --- | --- | --- | --- | --- | --- | --- | --- | --- | --- | --- |
| **Previous Occupation** | | | |  | |  | 6.016^b^ | 0.001 | | | |
| Civil servant | | | | 92（18.5%） | | 84.52±21.79 |  |  | | | |
| Worker | | | | 147（30.0%） | | 85.77±22.88 |  |  | | | |
| Farmer | | | | 121（27.5%） | | 95.35±16.40 |  |  | | | |
| Other | | | | 112（24.0%） | | 89.87±24.08 |  |  | | | |
| **Health Status** | | | |  | |  | 2.839^b^ | 0.024 | | | |
| Very good | | | | 58（12.3%） | | 94.29±23.15 |  |  | | | |
| Good | | | | 150（31.8%） | 89.03±20.40 |  |  | | |  |  |
| Fair | | | | 211（44.7%） | 87.62±21.63 |  |  | | |  |  |
| Poor | | | | 49（10.4%） | 86.00±24.15 |  |  | | |  |  |
| Very poor | | | | 4（0.8%） | 115.75±12.18 |  |  | | |  |  |
| **Willingness for Mutual Aid** | | | |  |  | 4.224^a^ | ＜0.001 | | |  |  |
| Yes | | | | 348（73.7%） | 91.45±20.41 |  |  | | |  |  |
| No | | | | 124（26.3%） | 81.97±24.16 |  |  | | |  |  |
| **Convenience of Going Out** | | | |  |  | -3.022^a^ | 0.003 | | |  |  |
| Yes | | | | 357（75.6%） | 87.25±22.26 |  |  | | |  |  |
| No | | | | 115（24.4%） | 94.26±19.61 |  |  | | |  |  |
| **Number of Children** | | |  |  | 5.670^b^ | 0.004 | |  |  |  |  |
| ≤1 | | | 130（25.9%） | 83.54±24.32 |  |  | |  |  |  |  |
| 2 | | | 174（37.6%） | 90.69±20.35 |  |  | |  |  |  |  |
| ≥3 | | | 168（36.6%） | 91.35±20.67 |  |  | |  |  |  |  |
| **No Caregiver** | | |  |  | -3.197^a^ | 0.001 | |  |  |  |  |
| Yes | | | 320（67.8%） | 86.76±21.66 |  |  | |  |  |  |  |
| No | | | 152（32.2%） | 93.57±21.55 |  |  | |  |  |  |  |
| **Neighbour's Care** | | |  |  | 2.398^a^ | 0.017 | |  |  |  |  |
| Yes | | | 355（75.2%） | 90.33±20.96 |  |  | |  |  |  |  |
| No | | | 117（24.8%） | 84.78±23.92 |  |  | |  |  |  |  |
| **Subsidy from Children** | | |  |  | 3.746^a^ | ＜0.001 | |  |  |  |  |
| Yes | | | 172（36.4%） | 93.86±16.92 |  |  | |  |  |  |  |
| No | | | 300（63.6%） | 86.14±23.78 |  |  | |  |  |  |  |
| **Type of medical insurance** | |  |  | 12.345^b^ | ＜0.001 |  |  |  |  |  |  |
| Urban employees insurance | | 231（46.5%） | 84.45±22.39 |  |  |  |  |  |  |  |  |
| Urban residents insurance | | 104（23.0%） | 92.74±21.10 |  |  |  |  |  |  |  |  |
| New rural cooperative medical insurance | | 111（25.7%） | 97.20±16.11 |  |  |  |  |  |  |  |  |
| Other insurance | | 26（4.9%） | 78.69±27.15 |  |  |  |  |  |  |  |  |
| **Retirement Pension** | |  |  | -2.871^a^ | 0.004 |  |  |  |  |  |  |
| Yes | | 354（73.6%） | 87.30±22.60 |  |  |  |  |  |  |  |  |
| No | | 118（26.4%） | 93.92±18.59 |  |  |  |  |  |  |  |  |
| **Medical Institution Care** | |  |  | 2.490^a^ | 0.013 |  |  |  |  |  |  |
| Yes | | 201（42.6%） | 91.85±20.91 |  |  |  |  |  |  |  |  |
| No | | 271（57.4%） | 86.81±22.30 |  |  |  |  |  |  |  |  |
| **Volunteer Service Care** | |  |  | 3.204^a^ | 0.001 |  |  |  |  |  |  |
| Yes | | 120（25.4%） | 94.42±21.42 |  |  |  |  |  |  |  |  |
| No | | 352（74.6%） | 87.09±21.70 |  |  |  |  |  |  |  |  |
| **Government Care** | |  |  | 2.202^a^ | 0.028 |  |  |  |  |  |  |
| Yes | | 314（66.5%） | 90.52±21.23 |  |  |  |  |  |  |  |  |
| No | | 158（33.5%） | 85.85±22.74 |  |  |  |  |  |  |  |  |
| **City Size** | |  |  | -3.610^a^ | ＜0.001 |  |  |  |  |  |  |
| First- and Second-tier Cities | | 241（51.1%） | 85.45±22.81 |  |  |  |  |  |  |  |  |
| Third- and Fourth-tier Cities | | 231（48.9%） | 92.61±20.17 |  |  |  |  |  |  |  |  |

Note: ^a^ represents t-value. ^b^ represents F-value.

Appendix VII.

Evaluation of Regression Model Performance Based on Different Algorithms (n=142)

| **Model** | **Mean Absolute Error** | **Mean Squared Error** | **Root Mean Squared Error** | **Coefficient of Determination** |
| --- | --- | --- | --- | --- |
| Multiple Linear Regression | 14.942 | 387.651 | 19.689 | 0.230 |
| Random Forest Regression | 16.613 | 472.079 | 21.727 | 0.062 |
| Support Vector Regression | 16.358 | 425.342 | 20.624 | 0.155 |
